# Evaluation of the Anxiolytic and Anti-Epileptogenic Potential of *Lactuca Serriola* Seed Using Pentylenetetrazol-Induced Kindling in Mice and Metabolic Profiling of Its Bioactive Extract

**DOI:** 10.3390/antiox11112232

**Published:** 2022-11-12

**Authors:** Muhammad Ihsan Ullah, Rukhsana Anwar, Shahzad Kamran, Bazgha Gul, Sameh S. Elhady, Fadia S. Youssef

**Affiliations:** 1 Punjab University College of Pharmacy, University of the Punjab, Lahore 54000, Pakistan; 2 Institute of Pharmacy, Lahore College for Women University, Lahore 54000, Pakistan; 3 Department of Natural Products, Faculty of Pharmacy, King Abdulaziz University, Jeddah 21589, Saudi Arabia; 4 Department of Pharmacognosy, Faculty of Pharmacy, Ain-Shams University, Abbasia, Cairo 11566, Egypt

**Keywords:** *Lactuca serriola*, anxiolytic, anticonvulsant, epilepsy, Industrial development, drug discovery, molecular docking

## Abstract

This study aimed to assess the potential of *Lactuca serriola* (Asteraceae) seed *n*-hexane, chloroform, methanol, and aqueous extracts as anticonvulsant, sedative, anticonvulsant and antiepileptic agents in Swiss albino mice. Different doses of each extract were evaluated for the anxiolytic potential using the hole-board, the elevated plus maze and the light/dark test. A phenobarbitone-induced sleep test was employed for the evaluation of sedative potential. Acute anticonvulsant activity was evaluated by picrotoxin and strychnine-induced convulsion models. All extracts significantly reduced the number of head dips where *n*-hexane extract (400 mg/kg) showed 96.34% reduction in the tendency of head dipping when compared with the control. Mice treated with extracts preferred elevated plus maze open arms and were shown to lack open arms evasion, especially *n*-hexane extract (400 mg/kg)—which showed 456.14%—increased the duration of open arm stay with the respective control group. By reducing sleep latency and greatly lengthening sleep duration, *L. serriola* enhanced the effects of barbiturate-induced sleep. A significant increase in convulsion latency and decrease in convulsions induced by picrotoxin and strychnine duration was observed in all extract-treated groups. All the extracts exhibited anti-epileptogenic potential as the seizure score in pentylenetetrazol (PTZ)-induced kindling in mice was reduced significantly. Maximum protection was afforded by chloroform extract that reduced the seizure score by 79.93% compared with the PTZ group. Chloroform executed antioxidant effect by elevating super oxide dismutase (SOD) by 126%, catalase (CAT) by 83.53%, total glutathione (tGSH) by 149%, and reducing malondialdhyde (MDA) levels by 36.49% in the brain tissues that is further consolidated by histopathological examination. Metabolic profiling of the most active chloroform extract using Gas chromatography coupled with mass showed the presence of 16 compounds. This anti-epileptic activity was further confirmed via in silico molecular modelling studies in the active site Gamma-aminobutyric acid aminotransferase (GABA-AT) where all of the tested metabolites illustrated a potent inhibitory potential towards GABA-AT with hexadecanoic acid, 15-methyl-, methyl ester followed by octadecanoic acid, methyl ester showed the best fitting. The results indicated the possible anxiolytic and anti-epileptogenic potential of the plant and further consolidated the ethnopharmacological use of *L. serriola* seeds.

## 1. Introduction

Anxiety and depression are characterized as psychological situations resulting in harmful emotional experiences [1]. WHO states that 322 million people worldwide are affected by anxiety, and meanwhile, 70 million people are experiencing epilepsy [2]. Epilepsy is a chronic neurological disorder that is characterized by an excessive burst of electrical discharge in different central nervous system (CNS) parts that concomitantly results in abrupt epileptic seizure onset and is the second most common chronic neurological condition seen by neurologists [3,4]. These seizures lead to alterations in behavior, sensations, muscle twitching, and sometimes loss of awareness and other life disabilities [5].

Available treatments for stress, anxiety, and epileptic disorders depend heavily on hormones and neurotransmitters. Synthetic medicines such as benzodiazepines are commonly prescribed for anxiety disorders [6]. Epilepsy is managed by conventional medications and newer antiepileptic drugs, but they are greatly linked to drug resistance and epilepsy-related disorders including cognitive impairment and depression because of uncontrolled seizure activity [7]. The suboptimal effectiveness and side effects of these medications provoked the search for new treatment options in this field [8].

This encourages the search for alternative treatment options including herbal medications [9,10]. Natural remedies showed better efficacy and higher safety profiles compared with the synthetic drugs [11,12]. Numerous medicinal plants are recognized for anxiolytic and anticonvulsant activities, and these medicinal plants serve as a source of valuable medications that can be easily provided and less expensive, and hence useful in improving these disorders [13].

*Lactuca serriola* L. (Asteraceae) is commonly known as prickly lettuce and is traditionally used for sedative–hypnotic and anxiolytic purposes [14]. Its traditional use for headache, bronchitis, cough, inflammation, and ophthalmic purposes was also reported [15]. The plant is also used for human nutrition purposes [16]. The whole plant is rich in a milky sap that flows freely from wounds. The sap contains ‘lactucarium’, which is used for antispasmodic and narcotic purposes. It is also used for the treatment of insomnia, anxiety, neuroses, and hyperactivity in children [17]. It was investigated pharmacologically for sedative, antioxidant, hepatoprotective, anti-inflammatory, and anti-carcinogenic activities [18,19]. Its phytochemical analysis previously revealed the presence of alkaloids, phenolic contents lactucin, lactucone, lactucic acids, lactucopicrin, sesquiterpene esters, vitamins, oxalic acid, *β*-carotene, and iron. The most abundant constituents isolated from *L. serriola* are lupeol, lupeol acetate, oleanans, *α*-amyrin, and *β*-amyrin [20,21,22].

Although *L. serriola* was extensively used in traditional medications as an anxiolytic agent, its anxiolytic and anti-epileptogenic potential have not been studied yet. Thus, this study aimed to investigate the anti-anxiety, anticonvulsant, and anti-epileptogenic activity of different *L. serriola* seed extracts in vivo using different models for the first time. The phenobarbitone-induced sleep test was conducted to examine the sedative properties of the drug. The anticonvulsant activity was assessed using picrotoxin and strychnine induced convulsion in mice whereas the anti-epileptogenic potential was evaluated using pentylenetetrazol-induced epileptic seizure in kindling mice model. Different biochemical parameters as super oxide dismutase (SOD), catalase (CAT), total glutathione (tGSH), and malondialdhyde (MDA) levels in the brain tissues were analyzed as antioxidants biomarkers that were further consolidated by histopathological examination. Additionally, the bioactive fractions were subjected to total phenolic content and total flavonoid content evaluation, whereas metabolic profiling of the chloroform extract was performed using Gas chromatographic analysis coupled with mass spectrometry (GC/MS). Additionally, in silico molecular docking studies of the main identified phytoconstituents from *Lactuca serriola* seed chloroform extract was performed in the active site of Gamma-aminobutyric acid aminotransferase (GABA-AT) aiming to explore its probable mode of action as antiepileptic drug. 

## 2. Materials and Methods

### 2.1. Plant Material

The seeds of *Lactuca serriola* L. (Asteraceae) were purchased from an herbal dealer in Lahore and were identified and authenticated by a botanist from the Botany department, Government College University (GCU) Lahore. Voucher number of GC. Herb. Bot. 3325 was allotted and the specimen was preserved in the departmental herbarium for future reference. 

### 2.2. Preparation of the Different Plant Extracts

To prepare the different plant extracts, *Lectuca serriola* (*L. serriola*) dried seeds were first ground into coarse size powder and then 1 kg of the powder was macerated for seven days at room temperature in *n*-hexane, chloroform, methanol, and distilled water in a respective manner for a sequential extraction process to prepare *n*-hexane, chloroform, methanol, and aqueous extracts, respectively. Five liters of each solvent were used for extraction; then after extraction, the solvents were evaporated at 45 °C under reduced pressure using a rotary evaporator followed by lyophilization to obtain dried lyophilized extracts. The dried extracts obtained were labeled after sealing in glass jars and were placed at 4 °C in the refrigerator. The percentage yield was calculated as:

%yield =weight of the obtained extract (g)/weight of plant material (g)×100

The yield of *L. serriola n*-hexane, chloroform, methanol, and aqueous extracts was calculated as 11%, 22%, 10%, and 12%, respectively.

### 2.3. In Vivo Biological Evaluation of Different L. serriola Dried Seeds Extracts

#### 2.3.1. Experimental Animals

The study was performed on male Swiss albino mice about 20–25 g supplied by Punjab University College of Pharmacy, University of the Punjab, Lahore. The standard conditions of temperature (24 ± 2 °C) and humidity (55–65%) were maintained with 12 h light-dark rotation in the animal house of Punjab University College of Pharmacy, PU, Lahore. The mice were having easy access to food and water *ad-libitum*. They were acclimatized to the laboratory environment seven days before the experimentation. The experimental protocols and procedures to be employed in the study was approved by the Institutional Ethics Review Board of the University with letter number D/243/FIMS.

#### 2.3.2. Acute Toxicity Study

*L. serriola* extracts were evaluated for acute toxicity study according to OECD 2001 Guidelines. The mice were divided into five groups with three mice in each group. They were kept on fasting for an overnight period without water for 3–4 h before treatment with the different extract. Normal saline was administered to the control group while extract-treated groups were administered with extract at doses of 5, 50, 300, and 2000 mg per kg body weight by oral guvage. The extracts were dissolved in a vehicle (3% tween 20) before administration. They were observed continuously for 4 h to detect toxicity signs such as tremors, sedation, ataxia, convulsions, hypnosis, and muscle spasm. The mice were further kept under observation for 48 h to detect any mortality. For the assessment of delayed toxicity, the observation was continued for further 14 days.

#### 2.3.3. In Vivo Anxiolytic Activity Evaluation of Different *L. serriola* Dried Seeds Extracts

To evaluate the anxiolytic activity of different *L. serriola* dried seeds extracts, the mice were distributed into four main groups, with 30 mice in each group. The first group is the *n*-hexane group, the second group is the chloroform group, and the third group is the methanol group whereas the fourth group is the aqueous group. Each of these groups was further sub-divided into five sub-groups (*n* = 6). Group I, normal control; Group II, standard, group receiving diazepam 1 mg/kg orally, whereas groups III-V, the animals were treated with the respective extract orally at doses of 300, 400 and 500 mg/kg body weight, respectively. The extracts were dissolved in a vehicle (3% tween 20) before administration.

#### Hole-Board Test

The hole-board apparatus was used for the assessment of anxiolytic and CNS depressant activity. The apparatus contains sixteen equidistant holes on a black sheet that was placed on a transparent poly acrylic rectangular box. The floor of the box was placed 15 cm above the ground. The mice under study were orally treated with vehicle and diazepam 1 mg/kg, and each extract of *L. serriola* at a dose of 300, 400, and 500 mg/kg was administered 1 h before recording the number of head dips. Each mouse was placed at the center of the apparatus and was allowed to explore the apparatus liberally for 5 min, and the number of head dips was counted [23]. A head dip was counted only when both eyes of the mouse disappeared in the hole [24]. The apparatus was washed completely with 10% ethanol solution for the next mice.

##### Elevated Plus Maze Test

The elevated plus maze (EPM) test was used for the assessment of anxiety, stress, motor coordination, and exploratory behavior of rodents. The apparatus contains 4 arms, two open and two closed, 49 cm in length and 10 cm broad, arranged in such a way that both arms are opposite to each other in the arrangement and the maze was placed 50 cm above the floor. The mice under study were administered with vehicle, diazepam 1 mg/kg, and with each extract of *L. serriola* at dosses of 300, 400, and 500 mg/kg orally just 1 h before the test. Each mouse was placed on the platform of EPM and the time spent and the number of entries in open arms was noted for five minutes. An entry was noted when the mouse placed all four paws in the arm [23]. The EPM apparatus was cleaned with 10% ethanol solution after the performance of the test, for the next mice.

##### Light/Dark Box Test

The light–dark test was also employed for the assessment of anxiolytic activity of investigational substances. The apparatus consists of a box divided into two compartments, a bright compartment painted white and a dark compartment painted dark. The two compartments are linked through a small opening at the center of both compartments. The mice were administered with vehicle, diazepam 1 mg/kg, and each extract of *L. serriola* dried seeds at dosses of 300, 400, and 500 mg/kg just 1 h before the test. Each mouse after treatment was placed in the center of the light box and observation was made for five minutes. The time spent by each mouse in the light compartment was recorded [25]. The mouse was returned to the cage after being removed from the box. The apparatus was washed thoroughly with 10% ethanol solution for the next mice.

#### 2.3.4. In Vivo Sedative Activity Evaluation of Different *L. serriola* Dried Seeds Extracts

The sedative activity of the *L. serriola* extracts was evaluated with a phenobarbitone-induced sleep test. For each extract of *L. serriola,* the mice were distributed into four groups (*n* = 30). The first group is the *n*-hexane group, the second group is the chloroform group, and the third group is the methanol group whereas the fourth group is the aqueous group. Each extract group was sub-divided further into 5 sub-groups I-V (*n* = 6). Group I was treated with vehicle; from group II-IV the animals were treated orally with the respective extract at doses of 300, 400, and 500 mg/kg body weight, respectively, whereas group V was i.p. administered with phenobarbitone at a dose of 100 mg/kg body weight. In addition, groups from I-IV were i.p. treated with phenobarbitone at a dose of 100 mg/kg body weight after 1 h of treatment with the different extracts. Different parameters were observed including the latency to onset of sleep and the duration of sleep [26].

#### 2.3.5. In Vivo Anti-Convulsive Activity Evaluation of Different *L. serriola* Dried Seeds Extracts

The anticonvulsant activity of *L. serriola* extracts was assessed through strychnine and picrotoxin-induced convulsions in mice in two separate experiments. The mice were distributed into four main groups, with 30 mice in each group. The first group is the *n*-hexane group, the second group is the chloroform group, and the third group is the methanol group whereas the fourth group was the aqueous group. Each of these groups was further sub-divided into five sub-groups (*n* = 6). Group I, normal control that received vehicle; Group II, standard, group receiving diazepam at a dose of 1 mg/kg as a standard drug against strychnine in the first experiment and the animals were treated with 2 mg/kg phenobarbitone as a standard drug against picrotoxin in the second experiment. In groups III-V, the animals were treated with the respective extract at doses f 100, 200 and 300 mg/kg body weight, respectively. All the groups after 30 min of treatments were intraperitoneally administered with 5 mg/kg of chemoconvulsant agents, strychnine, and picrotoxin, in the two experiments, respectively. The mice were considered protected if they did not show any convulsive activity after 30 min period. The mice showing convulsive activity were observed for latency to the onset of seizures and duration of seizures [27].

#### 2.3.6. In Vivo Anti-Epileptic Activity Evaluation of Different *L. serriola* Dried Seeds Extracts

The anti-epileptic activity of different *L. serriola* dried seeds extracts was assessed using pentylenetetrazol-induced kindling mice model. The mice were randomly divided into seven groups with six mice in each group. Group I was treated with vehicle; in group II, the animals were intraperitoneally treated with 35 mg/kg pentylenetetrazol (PTZ). In group III, the standard group, the animals were treated with 100 mg/kg of valproic acid whereas in groups IV-VII, the animals were treated with 300 mg/kg of each *n*-hexane, chloroform, methanol and aqueous extracts, respectively. PTZ was intraperitoneally administered 2 h after the administration of valproic acid (VA), and different *L. serriola* dried seeds extracts. 

##### Pentylenetetrazol (PTZ) Induced Kindling in Mice

PTZ was used intraperitoneally for induction of kindling in mice at a sub-convulsive dose of 35 mg /kg every day with a total of eleven doses. The observations were made for 1 h for seizure scoring in mice and lethality. Seizure scoring was assessed by applying the following Racine scale [28] where 0: no response, 1: ear and facial twitching, 2: myoclonic jerks (without rearing), 3: myoclonic jerks (with rearing), 4: turning over into a side position (clonic–tonic seizures), 5: turning over into the back position (generalized tonic-clonic convulsions) and 6: mortality. The mice under observation were supposed to be entirely kindled when they had received a total of eleven doses of PTZ, and exhibited stage (4 or 5) of seizure scoring on three successive stages. PTZ at a dose of 75 mg/kg was considered a challenge dose on the test day (26^th^ day) in kindled mice. All the treatment groups were tested with a challenge dose, which induced tonic-clonic convulsions, and lethality [29].

##### Evaluation of the Biochemical Parameters in Brain Tissue

For assessment of biochemical parameters, the mice were anesthetized with chloroform and decapitated for brain isolation. The isolated brains were rinsed in ice-cold normal saline solution. The brains after weighing were homogenized by tissue homogenizer in ice-cold phosphate buffer (pH 7.4.) [30].

##### Assessment of Catalase (CAT) Activity in Brain

CAT activity was assessed by the method previously reported by Sinha [31] with slight modifications. The tissue homogenate was added to 0.01 M phosphate buffer at pH 7.0, and 0.2 M H_2_O_2_. Dichromate acetic acid containing potassium dichromate (5%), and glacial acetic acid in a ratio of 1:3 was mixed with the reaction mixture to stop the reaction. The test tubes were heated for 10 min in a water bath. The absorbance was measured at 570 nm after cooling at room temperature. CAT activity in the homogenate was estimated from the calibration curve [32].

##### Assessment of Malondialdehyde (MDA) Level in Brain

Assessment of the MDA level was made with thiobarbituric assay (TBA) following the Ohkawa method with few modifications [33]. Brain samples were homogenized in KCl solution (1.15%) to prepare tissue homogenate (10%). Three replicates each composing of 8% sodium lauryl sulfate (0.2 mL), 20% acetic acid (1.5 mL), 0.8% thiobarbituric acid (1.5 mL), distilled water (0.3 mL), and tissue homogenate (0.5 mL) were prepared. In blank, KCl (1.15%) was added instead of tissue homogenate. The reaction mixture was incubated for 1 h at 98 ºC, and then 5 mL of *n*-butanol were added after cooling. Centrifugation was made for thirty min at 4000 rpm. The supernatant was separated and the absorbance was noted at 532 nm for the estimation of MDA level in µM/g tissue using the standard calibration curve [34].

##### Assessment of Superoxide Dismutase (SOD) Level in Brain

SOD levels in the brain tissues were estimated by applying the method previously described by Magnani et al. [35] with some modifications [34]. The brain sample was homogenized in 67 mM phosphate buffer at pH 7.4 in a ratio of 1:3 to prepare tissue homogenate. Three replicates each containing tris-HCl buffer (1 mL) and homogenate (50 µL) were prepared. Then 0.2 mM pyrogallol solution (1 mL) was added in sample and control tubes. The blank contained only tris-EDTA buffer with pH 8.2. The absorbance was noted at 532 nm by spectrophotometer after adding pyrogallol solution at 0 and 1 min intervals. SOD activity was assessed by the formula shown as:

% Inhibition of pyrogallol auto−oxidation = ΔA (test)/ΔA (control)×100

SOD activity (U/mL)=% inhibition of pyrogallol auto−oxidation / 50%

##### Assessment of Total Glutathione (tGSH) Activity in Brain

The estimation of tGSH activity in brain tissues was performed by the method previously reported by Sedlak and Lindsay with few modifications [36]. The brain issues were homogenized in 67 mM phosphate buffer with pH 7.4. Immediately 25% of TCA was added to precipitate the homogenate. The reaction mixture was centrifuged at 4 °C for 40 min at 4200 rpm. To the supernatant, 200 mM Tris-HCl buffer containing 0.2 M EDTA pH 7.5, 10 mM DTNB and methanol (7.9 mL) were added in respective manner. 67 mM Phosphate buffer of pH 7.4 were added in the blank instead of the homogenate. The test tubes were vortexed and incubated at 37 °C in an oven for 30 min. The absorbance was measured at 412 nm by a spectrophotometer. tGSH standard curve was constructed by using different dilutions [32]. 

#### 2.3.7. Histopathological Examination

To assess the histological alterations in the frontal cortex and hippocampus, the brain tissues were isolated, cut, and then fixed instantaneously in formalin solution (10%) for 24 h period. The tissues were dehydrated and then fixed with paraffin. Then the tissues were cut at 5 µm thickness, and then eosin and hematoxylin dyes were applied for staining purposes according to the standard protocols. To prevent observer bias during evaluation, the slides were coded. The histological changes in extract treatment groups were observed under a light microscope. The surviving cells were denoted as round-shaped, cytoplasmic membrane-intact cells, without any nuclear condensation or distorted aspect. The acidophilus neuron, identified by intense cytoplasmic eosinophilia accompanied by chromatin dispersion with loss of nuclear membrane integrity, was perceived as the marker for irreversible neuronal damage at the cellular level [37].

### 2.4. Phytochemical Analysis of Different L. serriola Dried Seeds Extracts

#### 2.4.1. Estimation of Total Phenol Content

The Folin–Ciocalteu method was employed for the evaluation of the total phenol content of *L. serriola* extracts [38]. The total phenol contents in each extract were estimated from the linear regression equation by plotting the gallic acid calibration curve. The results were represented as gallic acid (mg) equivalents per (g) dried extract.

#### 2.4.2. Estimation of Total Flavonoid Content

Aluminum chloride colorimetric method was utilized for the assessments of total flavonoid content in *L. serriola* extracts [39]. Quercetin standard curve was plotted for linear regression equation. The values were described as quercetin mg equivalents per gram of the dried extract.

#### 2.4.3. Metabolic Profiling of *L. serriola* Dried Seeds Chloroform Extract by GC/MS

The phytoconstituents available in the most bioactive chloroform extract were estimated by GC–MS analysis using the area normalization method. The SUPLECO column (DB5-MS 30 m × 0.25 mm × 0.25 um) was run at a 1.22 mL/ min flow rate while using Helium as a carrier gas. The preparation of the solution of the sample was made by dissolving 2 mg of *L. serriola* chloroform extract in methanol (10 mL). The oven starts temperature was 80 °C, which was increased to 280 °C. The experimental sample (1 μL) was injected in splitless mode in GC-MS by maintaining the injector temperature at 250 °C, whereas the temperature of the ion source was fixed at 280 °C. The sample total run time was 29 min. The constituents were identified based on Wiley Registry of Mass Spectral Data 8th edition, NIST Mass Spectral Library (December 2011), and previously reported literature [40,41,42].

### 2.5. Molecular Docking Study

Molecular docking experiment was performed for the main identified phytoconstituents from *Lactuca serriola* seed chloroform extract in the active site Gamma-aminobutyric acid aminotransferase (GABA-AT) (PDB code: 1OHV; 2.30 Å) downloaded from protein data bank [43].This was performed using Discovery Studio 4.5 (Accelrys Inc., San Diego, CA, USA) using C-Docker protocol and the binding energies (∆ G) were calculated from the equation as previously reported [42,44,45,46]. 

### 2.6. Statistical Analysis

The data were analyzed with Graph pad prism version 5. Analysis was made by one-way ANOVA, followed by post hoc Dunette’s test for comparison among different groups. The data were represented as mean ± SEM and *p* * < 0.05 was marked as significant.

## 3. Results

### 3.1. In Vivo Biological Evaluation of Different L. serriola Dried Seeds Extracts

#### 3.1.1. Acute Toxicity Study

The mice did not show any sign of toxicity or mortality after the administration of *L. serriola* extracts at doses of 5, 50, 300, or 2000 mg per kg oral dose

#### 3.1.2. In Vivo Anxiolytic Activity Evaluation of Different *L. serriola* Dried Seeds Extracts

##### Hole-Board Test and Effect on Number of Head Dips

**Figure 1:**
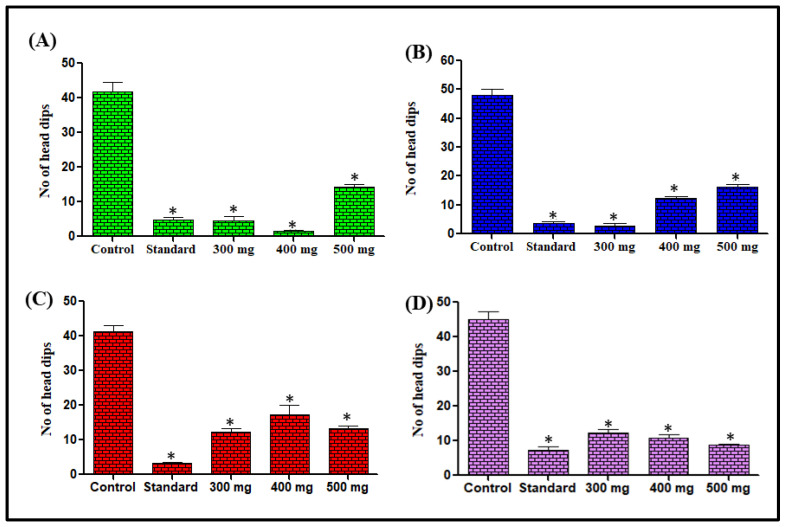
Effect of *n*-hexane (**A**), chloroform (**B**), methanol (**C**) and aqueous (**D**) extracts of *L. serriola* seeds on the number of head dips. Results were expressed as Mean ± SEM. A value of * *p* < 0.05 was considered statistically significant from the control group.

Mice treated with *L. serriola* extracts at doses of 300, 400, and 500 mg/kg body weight, the tendency of head dipping was decreased, as depicted in Figure 1, when compared with the control group. The most significant effect was observed for *n*-hexane extract at 400 mg/kg body weight, showing a 96.34% reduction in the tendency of head dipping whereas chloroform and methanol extracts at a dose of 300 mg/kg body weight revealed 94.17% and 70.07% reduction in the tendency of head dipping in comparison to the respective control groups. However, the aqueous extract at a dose of 500 mg/kg body weight showed 80.89% decrease in head dipping ability as compared to the control group (Figure 1).

##### Effect on Time Spent in the Elevated Plus Maze

**Figure 2:**
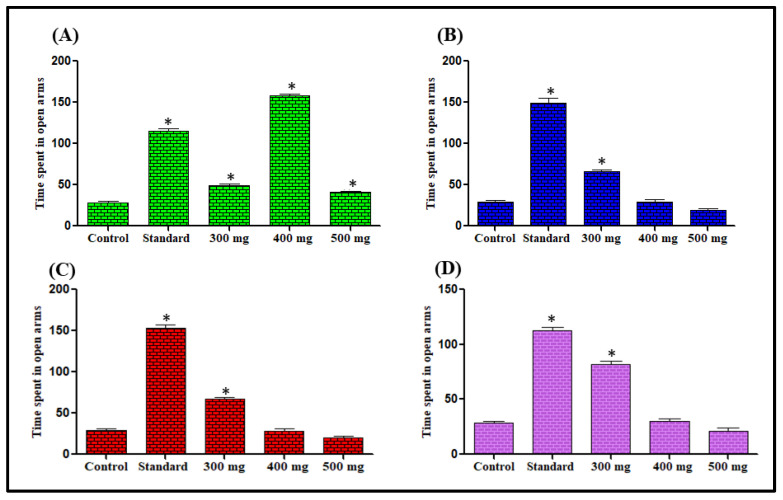
Effect of *n*-hexane (**A**), chloroform (**B**), methanol (**C**) and aqueous (**D**) extracts of *L. serriola* seeds on time spent in open arms in the elevated plus maze test. Results were expressed as Mean ± SEM. A value of * *p* < 0.05 was considered statistically significant from the control group.

**Figure 3:**
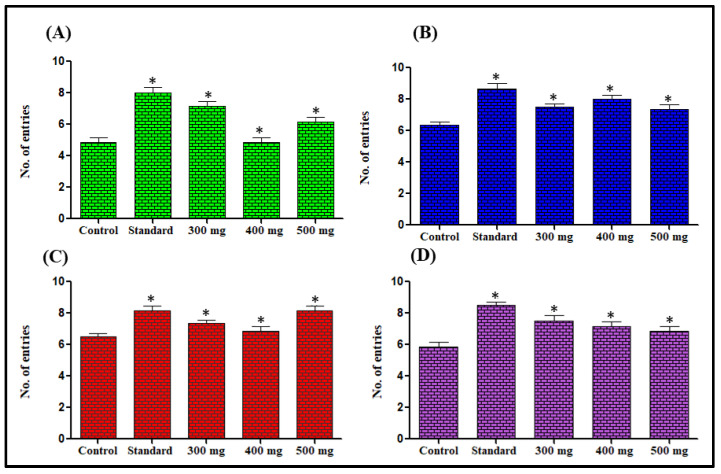
Effect of *n*-hexane (**A**), chloroform (**B**), methanol (**C**) and aqueous (**D**) extracts of *L. serriola* seeds on mice behavior in the elevated plus maze. Results were expressed as Mean ± SEM. A value of * *p* < 0.05 was considered statistically significant from the control group.

In this test, the mice administered with extracts showed a preference for open arms and were observed to be lacking in open arms avoidance. The control group mice constantly showed a preference for the closed arms of the maze considered comparatively protected portions. The administration of extracts at 300–500 mg/kg dose increased the duration of open arm stay significantly when compared with the control group. The most significant effects among the doses were found with *n*-hexane at 400 mg/kg dose, chloroform at 300 mg/kg dose, methanol 300 mg/kg dose and aqueous extract 300 mg/kg dose showing 456.14%, 126.90%, 191.58% and 185.57%, increased the duration of open arm stay respectively in comparison to the respective control groups (Figure 2). The number of entries for the open arms also increased. The most significant effects were observed for *n*-hexane at 300 mg/kg dose, chloroform at 400 mg/kg dose, methanol 500 mg/kg dose, and aqueous extract 300 mg/kg dose showing 47.92%, 18.48%, 24.62%, and 29.31% increase in the number of entries for the open arms respectively in comparison with the respective control groups (Figure 3).

##### Effect on Time Spent in Light Dark Box

**Figure 4:**
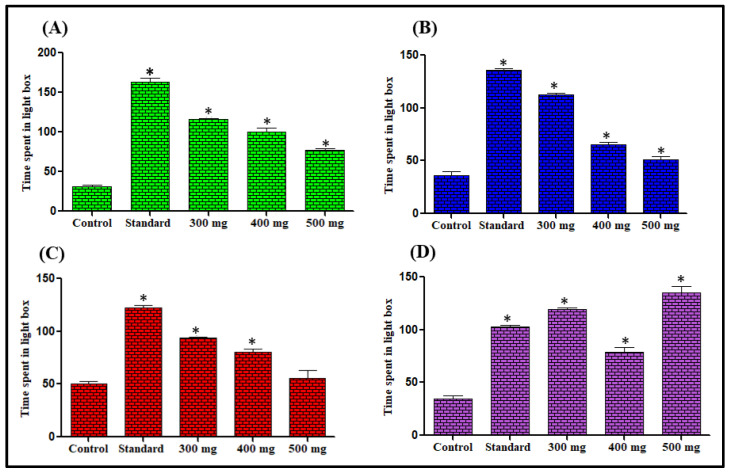
Effect of *n*-hexane (**A**), chloroform (**B**), methanol (**C**) and aqueous (**D**) extracts of *L. serriola* seeds on time spent (seconds) by mice in the light compartment. Results were expressed as Mean ± SEM. A value of * *p* < 0.05 was considered statistically significant from the control group.

The mice treated with different *L. serriola* extracts preferred the light compartment of the apparatus, while the vehicle-treated mice continually preferred the dark compartment of the light-dark model. The most significant effects were observed for *n*-hexane at 300 mg/kg dose, chloroform at 300 mg/kg dose, methanol, and aqueous extracts at 500 mg/kg dose showing 268.13%, 210.19%, 87.2%, and 288.22% increase in remaining in the light compartment respectively in comparison to the respective control groups (Figure 4).

#### 3.1.3. In Vivo Sedative Activity Evaluation of Different *L. serriola* Dried Seeds Extracts

Different *L. serriola* dried seed extracts enhanced the barbiturate-induced sleeping. The sleep latency was decreased significantly in a dose-dependent manner. The most significant effects of *n*-hexane, chloroform, methanol, and aqueous extracts were observed at 500 mg/kg dose showing 12.59%, 26.61%, 46.06% and 30.76% reduction in sleep latency, respectively in comparison with the respective control groups (Table 1). 

Additionally, the sleep duration was also significantly increased in a dose-dependent manner. The most significant effects of *n*-hexane, chloroform, methanol, and aqueous extracts were found at 500 mg/kg dose showing 53.37%, 47.59%, 54.52%, and 47.85% elevation in sleep duration, respectively (Table 2).

#### 3.1.4. In Vivo Anti-Convulsive Activity Evaluation of Different *L. serriola* Dried Seeds Extracts

The evaluation of the anti-convulsive activity of different *L. serriola* dried seeds extracts was performed using picrotoxin- and strychnine-induced convulsions. In picrotoxin-induced convulsions, *n*-hexane extract at 300 mg/kg and chloroform extract at 200 and 300 mg/kg completely protected the mice from seizure activity. Moreover, the seizure latency was increased, and seizure duration was decreased significantly in methanol and aqueous extracts, particularly at 300 mg/kg dose as the seizure latency was increased by 75.92% and 95.78%, respectively and the seizure duration was decreased by 43.60% and 47.21%, respectively in comparison to the control groups (Table 3).

Meanwhile, in strychnine-induced convulsions, all *L. serriola* extract, significantly increased the seizure latency (* *p* < 0.05) and decreased the seizure duration when compared with the control group. The most significant effects for *n*-hexane, chloroform, methanol, and aqueous extracts were observed at 300 mg/kg dose as the seizure latency increased by 95.65, 105.24%, 53.63%, and 45.56%, respectively, whereas the seizure duration decreased by 57.94%, 66.69%, 48.98%, and 58.97%, respectively, when compared with the control groups (Table 4).

#### 3.1.5. In Vivo Anti-Epileptic Activity Evaluation of Different *L. serriola* Dried Seeds Extracts

**Figure 5:**
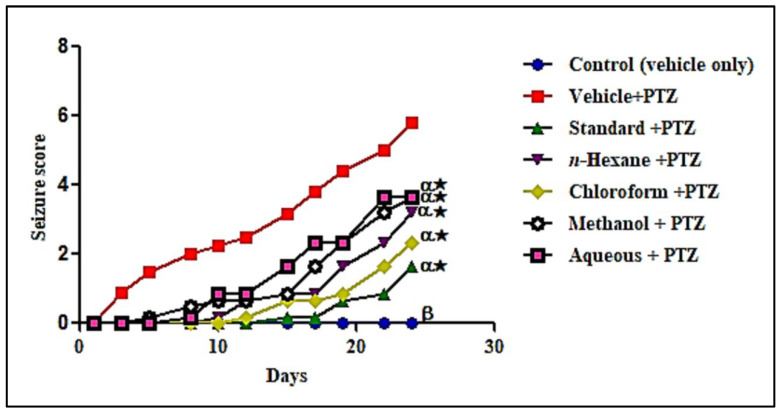
Effect of extracts of *L. serriola* (300 mg/kg) on seizure score in PTZ induced kindled mice. The standard is valproic acid (VA) (100 mg/kg); The treatment groups were compared with the PTZ groups presented as ‘α’ on the bar and the normal group compared with the PTZ group presented with ‘β’ on the bar. * Showed statistical difference at *p* < 0.05.

The seizure activity in mice was increased through the repeated administration of pentylenetetrazol (PTZ) at a sub-convulsive dose (35 mg/kg) with alternate-day dosing till 24 days. This seizure activity also caused stage 6 in the PTZ group only after crossing stage 5, with generalized clonic–tonic convulsions. Pretreatment with *L. serriola* extracts at 300 mg/kg dose orally significantly reduced the seizure score in comparison to the PTZ group. There was a decreased incidence and severity of seizure activity in extract-treated groups that showed efficient protection offered by *L. serriola* to the kindled mice. The maximum protection was found with chloroform extract that reduced the seizure score by 79.93% after eleven doses in comparison to the PTZ group. The *n*-hexane, methanol and aqueous extracts also showed significant effects in reducing the seizure score by 69.37%, 56.34% and 50.70%, respectively in comparison to the PTZ group (Figure 5). On the test day (26th day), a challenge dose of PTZ (75 mg per kg) was administered to the kindled mice. The seizure activity was also decreased with extract treatment. The more pronounced effect was observed with chloroform extract as the seizure latency was increased by 195.83% and seizure score was reduced by 42.09% when compared to the PTZ group. Additionally, the *n*-hexane, methanol, and aqueous extracts also significantly increased the seizure latency by 141.67%, 58.33% and 116.67%, respectively, and decreased the seizure scores by 36.35%, 34.78%, and 24.70%, respectively when compared to the control groups and the mortality rate was decreased significantly in most of extract-treated groups and the results were shown in Table 5.

##### Effect of Different *L. serriola* Dried Seeds Extracts on Various Oxidative Stress Markers in Brain Tissues

The MDA level was drastically increased in PTZ-induced kindling mice by 221.75% compared with the normal control group meanwhile pretreatment with different *L. serriola* dried seeds extracts significantly reduced MDA. The most significant effect was observed with *n*-hexane extract at a dose of 300 mg/kg showing 37.76% reduction when compared with the PTZ group. Furthermore, the chloroform, methanol, and aqueous extracts also significantly reduced the MDA by 36.49%, 6.72%, and 6.79%, respectively as compared to PTZ group. Additionally, CAT level in PTZ-induced kindling mice was decreased by 79.17% compared with the normal control group, whereas pretreatment with different *L. serriola* dried seeds extracts significantly enhanced its level. The most significant effect was observed with *n*-hexane extract, showing 89.41% elevation when compared to the PTZ group. Additionally, the chloroform, methanol, and aqueous extract also significantly restored the level of CAT by 83.53%, 54.82%, and 37.18%, respectively, when compared with PTZ group. Furthermore, the SOD level was decreased in the PTZ group by 66.32% compared with the normal control group, but pretreatment with different *L. serriola* dried seeds extracts significantly enhanced its level. The most significant effect was observed in the *n*-hexane group, showing 135.05% elevation when compared with PTZ group. Additionally, chloroform, methanol, and aqueous extracts significantly restored the level of SOD by 126%, 84.18%, and 50.87%, respectively, with regard to the PTZ group. The total GSH level was also dramatically decreased in the PTZ-treated group, showing a 74.31% reduction when compared with control, whereas pretreatment with different *L. serriola* dried seeds extracts significantly enhanced its level; the most significant effect was observed in the chloroform group, showing 149% elevation compared with the PTZ-treated group. However, *n*-hexane, methanol, and aqueous extract also significantly restored the total GSH by 135.14%, 67.57%, and 45.95%, respectively, compared with the PTZ-treated group (Table 6).

#### 3.1.6. Histopathological Examination

**Figure 6:**
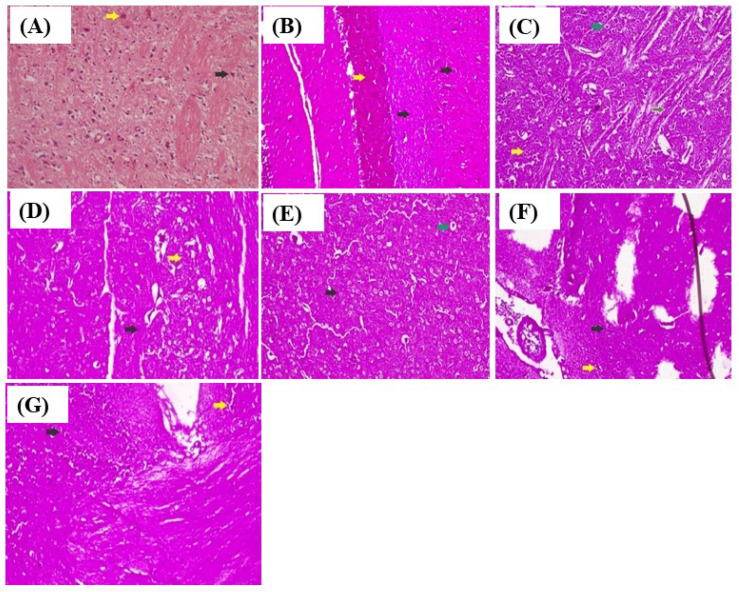
The morphology of frontal cortex and hippocampus neurons. (**A**) Control group, (**B**) PTZ group, (**C**) Standard group, (**D**) *L. serriola n*-hexane extract group, (**E**) *L. serriola* chloroform extract group, (**F**) *L. serriola* methanol extract group, and (**G**), *L. serriola* aqueous extract group.

Histopathological examination of the effect of *L. serriola* extracts on brain histology in PTZ-induced kindled mice revealed that *L. serriola* extracts effectively protected the neurons and normalized the histological alterations in PTZ-induced kindling mice. The histological examination illustrated normal neuronal organization and glial cells in the control group. Severe neuronal degeneration with the disorganization of neurons and gliosis was observed in the PTZ group. With *n*-hexane extract, fewer focal microglial reactions and mild neuronal degenerations were observed when compared with the PTZ group. In the chloroform extract-treated group, the neurons appeared to be normal with occasional neuronal degeneration and very few microglial reactions. In the methanol extract-treated group, moderate neuronal degenerations with focal microglial reactions were observed. In the aqueous extract-treated group, the degeneration of neurons with mild gliosis was observed in comparison with the PTZ group as shown in Figure 6. Hematoxylin and eosin stains showed glial cell infiltration, reduced number of shrunken and degenerated neurons, neuronal disorganization, and gliaosis in the mice’s frontal cortex and hippocampus. The black arrow points to the neuron, and the yellow arrow points to the glial cell in the control group (Figure 6A). The black arrow points to shrunken neurons, the yellow arrow points to degenerated glial cells, and the pink background points to gliaosis in the PTZ group (Figure 6B). The yellow arrow points to swollen glial cells, the green arrow points to oligodendro glial cells, and the brown arrow points to rosenthal fibers in the standard group (Figure 6C). The black arrow points to the shrunken neurons, the yellow arrow points to degenerated glial cells, and the pink background shows gliaosis in the *n*-hexane extract group (Figure 6D). The black arrow points to the neuron, the green arrow points to the oligodendro glial cell, and the pink background shows gliaosis in the chloroform extract group (Figure 6E). The black arrow points to the shrunken neurons, the yellow arrow points to degenerated glial cells, and the pink background shows gliaosis in the methanol extract group (Figure 6F). The black arrow points to the shrunken neurons, the yellow arrow points to degenerated glial cells, and the pink background shows gliaosis in the aqueous extract treatment group of *L. serriola* (Figure 6G).

### 3.2. Phytochemical Analysis of Different L. serriola Dried Seeds Extracts

#### 3.2.1. Estimation of Total Flavonoid and Phenol Contents

The total flavonoid content of *L. serriola* extracts was represented in mg quercetin equivalents/g. The chloroform extract contained more total flavonoid content estimated by181.41 mg QE/g followed by aqueous, methanol, and *n*-hexane extracts that showed 110.6, 84.41, and 46.08 mg QE/g, respectively. Furthermore, the total phenol content of *L. serriola* extracts was expressed in mg gallic acid equivalents/g. The methanol extract contained the maximum quantity of total phenol content as 562 mg GA/g followed by *n*-hexane, chloroform and the aqueous extracts showing 335, 35.37 and 10 mg GA equivalents/g, respectively.

#### 3.2.2. Metabolic Profiling of *L. serriola* Dried Seeds Chloroform Extract by GC/MS

**Figure 7:**
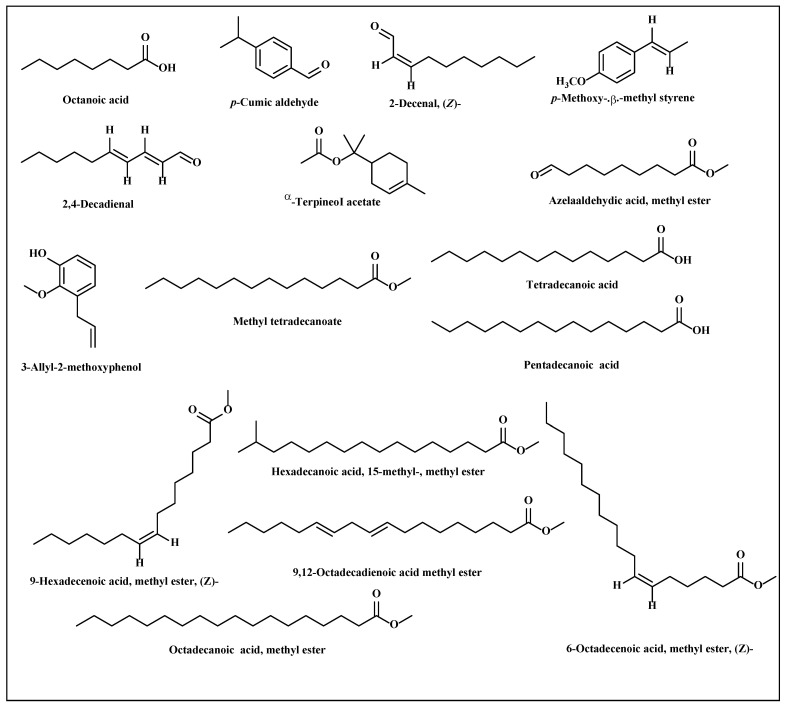
Chemical structures of the major identified compounds from *L. serriola* dried seeds chloroform extract using GC/MS.

The phytoconstituents available in the most bioactive chloroform extract were estimated using GC–MS analysis. A total of 16 compounds were identified in a reasonable quantity; the identified compounds as well as their percentage concentration were represented in Table 7. The highest quantity of 9, 12-octadecadienoic acid methyl ester (49.03%) was observed in the extract. Additionally, other compounds were detected with appreciable quantity represented by hexadecanoic acid, 15-methyl-, methyl ester (23.25%), octadecanoic acid, methyl ester (12.82%), 2,4-decadienal (4.93%), 3-allyl-2-methoxyphenyl (1.38%), tetradecanoic acid (1.34%), 6-octadecenoic acid, methyl ester (1.18%), and 2-decenal (1.10%). A scheme showing the chemical structures of the major identified compounds was illustrated in Figure 7.

### 3.3. Molecular Docking Study

In silico molecular docking studies of the main identified phytoconstituents from *Lactuca serriola* seed chloroform extract was performed aiming to explore its probable mode of action as antiepileptic drug. Basically, the decrease in the concentration of the inhibitory neurotransmitter γ-aminobutyric acid (GABA) in the brain resulted in neuronal excitation causing convulsions. This imbalance in neurotransmission can be reversed via the prohibition of γ-aminobutyric acid aminotransferase (GABA-AT) that plays a catalytic role during the conversion of GABA to the excitatory neurotransmitter L-glutamic acid [47]. Thus docking experiment was performed in the active site Gamma-aminobutyric acid aminotransferase (GABA-AT) (PDB code: 1OHV; 2.30 Å) downloaded from protein data bank by Discovery Studio 4.5 (Accelrys Inc., San Diego, CA, USA) using C-Docker protocol. Results displayed in Table 8 showed that all of the tested metabolites illustrated a potent inhibitory potential towards GABA-AT where hexadecanoic acid, 15-methyl-, methyl ester followed by octadecanoic acid, methyl ester showed the best fitting within the active sites with ∆G of −51.40 and −44.74 kcal/mol, respectively showing a superior activity in this regard compared to valaproic acid that revealed ∆G of −30.19 kcal/mol. 

**Figure 8:**
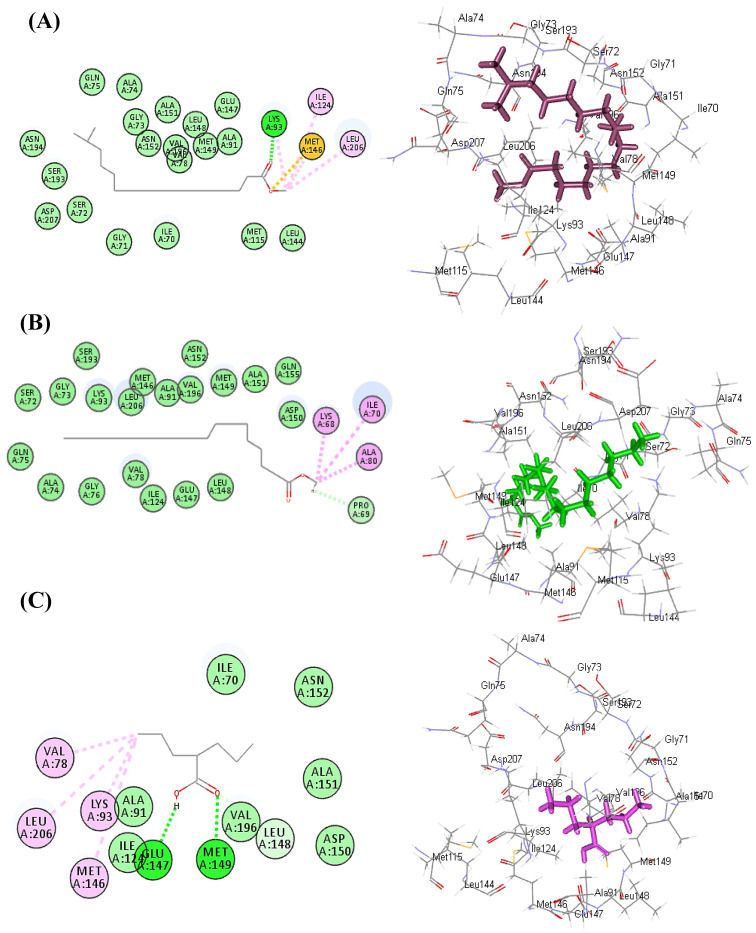
2D and 3D binding modes of hexadecanoic acid, 15-methyl-, methyl ester (**A**), octadecanoic acid, methyl ester (**B**) and valproic acid (**C**) within the active center of Gamma-aminobutyric acid aminotransferase (GABA-AT) (PDB code: 1OHV) using in silico studies.

Hexadecanoic acid, 15-methyl-, methyl ester forms one hydrogen bond with Lys93; two alkyl bonds with Ile124 and Leu206 in addition to sulfur bond with Met146 (Figure 8A). Regarding octadecanoic acid, methyl ester, it also showed three alkyl interactions with Lys58, Ile70, Ala80 in addition to one C-H bond with Pro69 (Figure 8B). Concerning valproic acid, it showed two conventional H-bonds with Glu147, Met149; four alkyl interactions with Val78, Leu206, Lys93, Met146 in addition to one C-H bond with Leu148 (Figure 8C). Thus, from the above illustrated values, it could be postulated that the antiepileptic potential of *Lactuca serriola* seed could be interpreted by the virtue of the synergistic action of its detected metabolites in inhibiting GABA-AT) causing a pronounced elevation in GABA brain concentration with the concomitant amelioration of epilepsy.

## 4. Discussion

Anxiety is considered a major psychiatric disorder with an enhanced socioeconomic burden on society where the number of patients in search of treatments is persistently rising, and thus anxiety-related disorders are escalating [48]. Moreover, epilepsy is a chronic neurological disorder that is characterized by persistent impulsive seizures. [49]. The kindling model is most commonly used for epileptogenic studies where sub-convulsant dosing of a chemical or an electrical stimulus in a repetitive and alternative manner may lead to progressive intensification of seizure activity [50].

Pentylenetetrazol (PTZ) is a GABA_A_ receptor antagonist that may cause the suppression of the inhibitory synapse functions that lead to the enhanced activity of neurons [51]. PTZ at higher doses induces acute and severe seizure activity, while the sequential administration of sub-convulsant dosing is used for kindling development in epileptic models. The resistance to the available anti-epileptic drugs may cause interference with the management of epileptic disorders, and thus worsening the patient’s condition.

Anxiety-related disorders are commonly managed by benzodiazepines. The anxiolytic and anti-convulsive activity of several plants was previously investigated to provide an alternative treatment choice for these disorders [52]. Different *L. serriola* dried seeds extracts were investigated for anxiolytic, CNS depressant, and anticonvulsant potential in this study. The hole–board test is a simple procedure to determine the emotional and anxiety-related responses of animals to unfamiliar conditions that were employed in the study [53]. The studies have reported that decreased number of head dips is the indicator for CNS depressant activity in animal models [54]. In the current study, the head dips were significantly decreased in comparison to the control group illustrating the CNS depressant action of *L. serriola*.

Elevated plus maze test is the most validated animal model for the evaluation of the anti-anxiety activity of substances under investigation. [55]. The extract-treated animals in this study showed anti-anxiety activity by exhibiting increased time spent in the open arm and the number of entries for open arms was also increased in comparison to the control groups. Previous studies revealed that the reliable parameter for anxiolytic activity assessment is the time spent in the light region than entries in the light region [56]. *L. serriola* each extract significantly enhanced the light region time spent in comparison to the control groups, and thus further confirming that *L. serriola* possesses anxiolytic properties. The action performed by anxiolytic drugs is by opening GABA-activated chloride ion channels to enhance the GABA response. So it may be hypothesized that *L. serriola* is showing action like benzodiazepines [57].

The phenobarbitone-induced sleep test was used for the evaluation of the sedative–hypnotic activity of investigational substances [58].The extract’s ability to enhance phenobarbitone-induced hypnosis is an additional support for the CNS depressant action of that extract. This CNS depressant activity is due to its effect on the central parameters associated with sleep regulation [59].The CNS depressant activity of the *L. serriola* was also consolidated by the ability of the extracts to enhance phenobarbitone-induced sleeping in a dose-dependent mode. The benzodiazepines were reported to exhibit anxiolytic activity at low doses and sedative-hypnotic activity at higher doses [60]. The sedative effect of *L. serriola* extracts at elevated doses suggested that the plant may resemble that of benzodiazepine receptor agonists such as diazepam. Anxiety and insomnia are often observed in patients with persistent epileptic disorders [61]. *L. serriola* might act as a supportive agent in these conditions due to its anxiolytic and hypnotic activities as diazepam [62].

Basically, GABA acts as the key inhibitory neurotransmitter in CNS and the anti-epileptic drugs cause an increased GABA-mediated inhibition in the brain. [63]. In the brain, picrotoxin induced convulsive activity by blocking chloride ions conductance through GABA_A_ receptors [64]. *L. serriola* extracts showed significant protection in the mice from picrotoxin-induced convulsive activity. It is proposed that *L. serriola* attenuated the picrotoxin-induced convulsions by increasing GABAergic neurotransmission. Moreover, the convulsive activity in the *n*-hexane and chloroform extract-treated mice was completely inhibited by the enhanced protection offered by these extracts. This particular protection might be due to the active anti-convulsant constituents being more extractable in these solvents. Glycine, also the inhibitory neurotransmitter has a vital role in the convulsive activity. Strychnine competitively inhibits glycine receptors postsynaptically located in the spine, brain stem, and higher centers. The inhibition of glycine receptors by strychnine would further lead to the blocking of inhibitory actions and enhanced motor neuron impulses leading to involuntary skeletal muscles spasm [27]. The current study illustrated that *L. serriola,* to a great extent, protected the animals from chemically induced seizures by delaying the onset and decreasing the duration of seizures that may be interpreted by the enhanced GABA-mediated inhibition triggered by the prevailing phytoconstituents. 

In the current study, *L. serriola* extracts significantly attenuated the seizure score in comparison to PTZ treated mice as the susceptibility to seizure activity was gradually enhanced in the PTZ group during the kindling process. There was a delay in the onset of seizures and the duration of seizures was also reduced in a significant manner, due to the neuroprotection offered by *L. serriola* extracts. During epileptic seizures there is a decrease in the antioxidant defense process and an increase in the number of free radicals in the brain resulting in an enhanced oxidative stress [65]. Herein, there was a significant decrease in ROS scavenging activity of cellular antioxidants (CAT, SOD, and total GSH) in the brain due to PTZ-induced seizure activity in addition to a marked increase in the level of MDA. Additionally, oxidative damage to the lipid membranes causes lipid peroxidation where in PTZ-induced kindling models the process of lipid peroxidation is enhanced in most cases as previously reported [66]. *L. serriola* dried seeds different extracts significantly restored the levels of SOD, CAT, and total GSH in the brain. and caused a significant reduction in the level of MDA when compared with the PTZ group, hence exhibiting neuroprotection in kindled mice owing to their. free radical scavenging activity [67]. 

Phytochemical evaluation of total flavonoid and phenol contents showed their presence in considerable amounts in the different extracts in addition to the existence of terpenoids in the chloroform extract. The flavonoids execute behavioral activities showing sedative and anti-anxiety potential in animal models [68]. Previous studies also reported the sedative and anti-anxiety activities of terpenoids on CNS [69]. From molecular docking studies, It is hypothesized that anxiolytic and anticonvulsant activity of *L. serriola* is due to synergistic action of its phytoconstituents in inhibiting GABA-AT) causing a pronounced elevation in GABA brain concentration with concomitant amelioration of epilepsy. Many studies reported the effectiveness of flavonoids and phenols in the management of epileptic seizures that is attributed to their antioxidant nature. These constituents either alone or in combination with antiepileptic drugs proved beneficial for managing epilepsy associated with comorbid conditions in animal models [70,71,72]. Many additional studies revealed that flavonoids due to structural similarity with benzodiazepines, execute their antiepileptic activities by modulating GABA_A_ chloride channel complexes [73]. Hence, flavonoids might reveal a modulating role to manage neurodegenerative disorders as via disrupting oxidative processes at cellular levels in the CNS [74].

The PTZ-induced seizures in the kindling models cause GABA level alterations in the brain. Since *L. serriola* showed activity against PTZ-induced seizures, it is supposed that its effect may be due to interference with GABA by increasing the GABA_A_ receptor activation because of available phytochemicals in it. That will assist the GABA-mediated chloride channel opening and offer symptomatic relief. 

## 5. Conclusions

In conclusion, *L. serriola* seed extracts showed strong anti-anxiety and anti-epileptic properties. In PTZ-induced kindling mice, *L. serriola* dried seed extracts demonstrated an outstanding CNS depressant and neuroprotection. They were able to minimize the burden of oxidative stress and boost the anti-oxidant mechanisms in the brain, providing significant neuronal protection. This was further confirmed via in silico molecular modelling studies in the active site Gamma-aminobutyric acid aminotransferase (GABA-AT) where all of the tested metabolites illustrated a potent inhibitory potential towards GABA-AT with hexadecanoic acid, 15-methyl-, methyl ester followed by octadecanoic acid, methyl ester showed the best fitting. This was additionally consolidated the traditional use of this plant to treat epilepsy and anxiety disorders. This potential activity could be interpreted by the virtue of the synergistic action of the essential phytochemicals predominating in the *L. serriola* dried seed extracts comprising flavonoids, phenol and terpenoids compounds evidenced by the phytochemical profiling and the GC/MS analysis. From the illustrated results, it can be hypothesized that *L. serriola* possesses anxiolytic and anti-epileptogenic potential through interfering with GABA neurotransmitters. Thus, *L. serriola* could serve as a possible antiepileptic treatment, however further in depth mechanistic studies followed by preclinical ones are highly recommended. In addition, phytochemical analysis of other polar extracts that should certain activity was highly recommended to be performed using LC-DAD-ESI-MS/MS as well as the docking study of the detected compounds.

## Figures and Tables

**Table 1:** Effect of different *L. serriola* seed extracts on the sleep onset (in minutes) in a phenobarbitone-induced sleep test in mice.

Extract	Control	Standard	300 mg/kg	400 mg/kg	500 mg/kg
*n*-Hexane	10.88 ± 0.04	10.06 ± 0.03 *	10.08 ± 0.04 *	9.98 ± 0.03 *	9.51 ± 0.06 *
Chloroform	11.35 ± 0.04	10.98 ± 0.04 *	9.76 ± 0.04 *	9.06 ± 0.03 *	8.33 ± 0.04 *
Methanol	10.03 ± 0.03	9.81 ± 0.03 *	8.95 ± 0.04 *	7.6 ± 0.05 *	5.41 ± 0.04 *
Aqueous	10.11 ± 0.03	9.96 ± 0.03 *	8.1 ± 0.02 *	7.55 ± 0.05 *	7 ± 0.03 *

Results were expressed as Mean ± SEM. A value of * *p* < 0.05 was considered statistically significant from the control group.

**Table 2:** Effect of different *L. serriola* seeds extracts on the sleep duration (in minutes) in a phenobarbitone-induced sleep test in mice.

Extract	Control	Standard	300 mg/kg	400 mg/kg	500 mg/kg
*n*-Hexane	189.8 ± 0.60	202.1 ± 0.83 *	241.6 ± 0.55 *	263.3 ± 0.88 *	291.1 ± 0.70 *
Chloroform	204.83 ± 0.47	214.33 ± 0.61 *	247 ± 0.57 *	273 ± 0.81 *	302.3 ± 0.71 *
Methanol	211.3 ± 0.61	190.66 ± 0.49 *	262.6 ± 0.71 *	304.1 ± 0.70 *	326.5 ± 1.08 *
Aqueous	197.5 ± 0.42	203.16 ± 0.47 *	260.16 ± 0.30 *	281.5 ± 0.67 *	292 ± 0.57 *

Results were expressed as Mean ± SEM. A value of * *p* < 0.05 was considered statistically significant from the control group.

**Table 3:** Effect of different *L. serriola* seeds extracts on picrotoxin-induced seizures in mice.

Treatment Groups	Seizure Latency	Seizure Duration	Mortality (Yes/No)
PCT	679 ± 3.22	213.3 ± 2.44	No
PCT + Phenobarbitone	-	-	No
PCT + *n*-hexane extract (100 mg)	1514 ± 2.95 *	110.8 ± 1.42 *	No
PCT + *n*-hexane extract (200 mg)	2011.1 ± 1.74 *	20.5 ± 1.17 *	No
PCT + *n*-hexane extract (300 mg)	-	-	No
PCT + chloroform extract (100 mg)	2253.8 ± 8.46 *	16.7 ± 2.15 *	No
PCT + chloroform extract (200 mg)	-	-	No
PCT + chloroform extract (300 mg)	-	-	No
PCT + methanol extract (100 mg)	821.5 ± 4.12 *	218.3 ± 2.23	No
PCT + methanol extract (200 mg)	1036.1 ± 6.73 *	184.8 ± 0.30 *	No
PCT + methanol extract (300 mg)	1194.5 ± 3.94 *	120.3 ± 3.06 *	No
PCT + aqueous extract (100 mg)	820.6 ± 4.06 *	214.6 ± 2.15	No
PCT + aqueous extract (200 mg)	1031.1 ± 5.26 *	168.1 ± 8.89 *	No
PCT + aqueous extract (300 mg)	1329.3.3 ± 8.22 *	112.6 ± 3.26 *	No

The seizure latency and duration of the seizure were expressed in seconds; Results were expressed as Mean ± SEM. A value of * *p* < 0.05 was considered statistically significant from the control group; Picrotoxin = PCT.

**Table 4:** Effects of different *L. serriola* seeds extracts on strychnine-induced seizures in mice.

Treatment Groups	Seizure Latency	Seizure Duration	Mortality (Yes/No)
STR	124.0 ± 0.93	48.33 ± 1.2	Yes
STR + Diazepam	214.5 ± 0.84 *	20.33 ± 1.87 *	Yes
STR + *n*-hexane extract (100 mg)	142.6 ± 0.88 *	31.00 ± 0.57 *	Yes
STR + *n*-hexane extract (200 mg)	178.3 ± 1.14 *	26.00 ± 0.44 *	Yes
STR + *n*-hexane extract (300 mg)	242.6 ± 0.84 *	20.33 ± 0.66 *	Yes
STR + chloroform extract (100 mg)	194.1 ± 2.73 *	20.50 ± 0.42 *	Yes
STR + chloroform extract (200 mg)	242.1 ± 0.94 *	17.83± 0.4 *	Yes
STR + chloroform extract (300 mg)	254.5 ± 1.05 *	16.10 ± 0.6 *	Yes
STR + methanol extract (100 mg)	147.1 ± 1.47 *	31.30 ± 0.66 *	Yes
STR + methanol extract (200 mg)	170.5 ± 0.76 *	28.83 ± 0.30 *	Yes
STR + methanol extract (300 mg)	190.5 ± 0.76 *	24.66 ± 0.49 *	Yes
STR + aqueous extract (100 mg)	153 ± 1.03 *	29.5 ± 0.67 *	Yes
STR + aqueous extract (200 mg)	162.6 ± 1.05 *	26.50 ± 0.67 *	Yes
STR + aqueous extract (300 mg)	180.5 ± 0.76 *	19.83 ± 0.60 *	Yes

The seizure latency and duration of the seizure were expressed in seconds; Results were expressed as Mean ± SEM. A value of * *p* < 0.05 was considered statistically significant from the control group; Strychnine = STR.

**Table 5:** Effect of different *L. serriola* seeds extracts (300 mg/kg) on seizure latency, intensity, and mortality in kindled mice with a challenge dose (75 mg/kg) of pentylenetetrazol (PTZ).

Treatment	Seizure Latency	Seizure Intensity	Mortality
PTZ only	240± 0.57	5.75 ± 0.28	5/6
Standard (VA)	680 ± 0.88 *	3.25 ± 0.28 *	1/6 *
*n*-Hexane	580 ± 1.76 *	3.66 ± 0.33 *	1/6 *
Chloroform	710 ± 1.20 *	3.33 ± 0.33 *	1/6 *
Methanol	380 ± 0.33 *	3.75 ± 0.28 *	3/6 *
Aqueous	520 ± 1.20 *	4.33 ± 0.33 *	3/6 *

The seizure latency WAS expressed in seconds; Results were expressed as Mean ± SEM. A value of * *p* < 0.05 was considered statistically significant from pentylenetetrazol (PTZ) group; valproic acid (VA) (100 mg/kg).

**Table 6:** Effect of different *L. serriola* dried seeds extracts (300 mg/kg) on various oxidative stress markers in brain tissues.

Treatment Group	MDA (µM/mg Tissue)	CAT (µM/mg Tissue)	SOD (U/mL)	Total GSH (mM/g)
Normal	8.51 ± 0.28	40.8 ± 0.44	61.76 ± 0.50	1.44 ± 0.00
PTZ	27.38 ± 0.08	8.50 ± 0.57	20.80 ± 0.19	0.37 ± 0.01
Standard	18.75 ± 0.23 *	16.6 ± 0.60 *	50.88 ± 1.56 *	1.01 ± 0.01 *
*n*-Hexane	17.04 ± 0.39 *	16.6 ± 0.88 *	48.89 ± 2.51 *	0.87 ± 0.01 *
Chloroform	17.39 ± 0.06 *	15.6 ± 0.92 *	47.01 ± 0.97 *	0.92 ± 0.00 *
Methanol	25.54 ± 0.05 *	13.16 ± 0.44 *	38.31 ± 0.39 *	0.62 ± 0.00 *
Aqueous	25.52 ± 0.18 *	11.66 ± 0.44 *	31.38 ± 0.62 *	0.54 ± 0.00 *

The data were expressed as Mean ± SEM (*n* = 6). A value of * *p* < 0.05 was considered statistically significant from pentylenetetrazol (PTZ) group.

**Table 7:** Metabolic profiling of *L. serriola* dried seeds chloroform extract using GC/MS analysis.

No.	Name	Retention Time	Retention Index	Concentration (%)
1	Octanoic acid	7.47	1179	0.63
2	*p*-Cumic aldehyde	9.20	1239	0.35
3	2-Decenal, (*Z*)-	9.66	1250	l. 10
4	*p*-Methoxy-*.beta*.-methyl styrene	10.28	1303	0.38
5	2,4-Decadienal	10.44	1309	4.93
6	α-Terpineol acetate	I 1.85	1354	0.73
7	3-Allyl-2-methoxyphenol	12.01	1392	1.38
8	Azelaaldehydic acid, methyl ester	13.81	1436	0.30
9	Methyl tetradecanoate	20.57	1706	0.84
10	Tetradecanoic acid	21.37	1770	1.34
11	Pentadecanoic acid	23.45	1857	0.45
12	9-Hexadecenoic acid, methyl ester, (*Z*)-	24.32	1879	0.37
13	Hexadecanoic acid, 15-methyl-, methyl ester	24.78	1996	23.25
14	9,12-octadecadienoic acid methyl ester	28.10	2094	49.03
15	6-Octadecenoic acid, methyl ester, (*Z*)-	28.30	2104	1.18
16	Octadecanoic acid, methyl ester	28.64	2112	12.82
	Total identified compounds			97.98%

**Table 8:** Free binding energies (kcal/mol) of major compounds in the active site Gamma-aminobutyric acid aminotransferase (GABA-AT) (PDB code: 1OHV) using in silico studies.

Compound	GABA-AT (1OHV)	Number of Formed Hydrogen Bonds and C-H Bonds	Number of Formed Alkyl and π-Alkyl Bonds
2,4-Decadienal	−20.37	3; Gln75, Gly76	-
2-Decenal, (*Z*)-	−21.41	2; Gly76, Lys93	-
3-Allyl-2-methoxyphenol	−14.92	2; Met149	4; Leu206, Val196, Val78, Ala91
6-Octadecenoic acid, methyl ester, (*Z*)-	−29.92	3; Lys93, Glu111, Asp207	-
9,12-octadecadienoic acid methyl ester	−15.46	-	3; Lys93, Leu206
9-Hexadecenoic acid, methyl ester, (*Z*)-	−26.32	3; Met448, Asp150, Ala151	1; Ile70
α-TerpineoI acetate	−0.69	1; Ala151	7; Val78, Val196, Leu206, Ala91, Ile70
Azelaaldehydic acid, methyl ester	−36.09	2; Asn152, Lys193	-
Hexadecanoic acid, 15-methyl-, methyl ester	−51.40	1; Lys93	2; Ile124, Leu206
Methyl tetradecanoate	−43.56	2; Asn152, Lys93	1; Leu206
Octadecanoic acid, methyl ester	−44.74	1; Pro69	3; Lys58, Ile70, Ala80
Octanoic acid	−33.64	2; Asn152, Gln155	-
*p*-Cumic aldehyde	−24.44	-	5; Val78, Val196, Ala91, Ile70
*p*-Methoxy-*.beta*.-methyl styrene	−11.04	3; Gln155, Ile70, Ala151	2; Val196, Ile70
Pentadecanoic acid	−44.01	2; Lys93, Asn194	-
Tetradecanoic acid	−42.94	2; Lys93, Asn194	-
Valproic acid	−30.19	3; Glu147, Met149, Leu148	4; Val78, Leu206, Lys93, Met146
Co-crystalized ligand	−7.74	2; Glu147, Met149	11; Val78, Val196, Ala91, Ile70, Leu206

## Data Availability

Data are available in the manuscript.
